# Searching for Low Probability Opening Events in a DNA Sliding Clamp

**DOI:** 10.3390/life12020261

**Published:** 2022-02-09

**Authors:** Reza Esmaeeli, Benedict Andal, Alberto Perez

**Affiliations:** Quantum Theory Project, Department of Chemistry, University of Florida, Gainesville, FL 32611, USA; reza.esmaeeli@chem.ufl.edu (R.E.); andalb@ufl.edu (B.A.)

**Keywords:** MELD, protein-DNA, molecular dynamics, REMD, coarse-grained

## Abstract

The β subunit of *E. coli* DNA polymererase III is a DNA sliding clamp associated with increasing the processivity of DNA synthesis. In its free form, it is a circular homodimer structure that can accomodate double-stranded DNA in a nonspecific manner. An open state of the clamp must be accessible before loading the DNA. The opening mechanism is still a matter of debate, as is the effect of bound DNA on opening/closing kinetics. We use a combination of atomistic, coarse-grained, and enhanced sampling strategies in both explicit and implicit solvents to identify opening events in the sliding clamp. Such simulations of large nucleic acid and their complexes are becoming available and are being driven by improvements in force fields and the creation of faster computers. Different models support alternative opening mechanisms, either through an in-plane or out-of-plane opening event. We further note some of the current limitations, despite advances, in modeling these highly charged systems with implicit solvent.

## 1. Introduction

The last 15 years have seen a dramatic increase in sampling ability, which has been driven by the optimization of software [1,2,3,4] as well as access to more efficient computer architectures [5,6]. Accessing longer timescales has helped with the identification and correction of force field deficiencies [7] leading to stable behavior on the millisecond timescale [8] using newer force fields [9,10]. Community efforts have served to identify best practices for simulation techniques [11,12,13] for free-DNA simulations, leading to a deeper mechanistic insight into free DNA behavior beyond Calladine’s rules [14]. Success with atomistic simulations has opened the door to more investigations of complex molecular systems involving DNA, such as stuiesy of minicircles, RNA-DNA chimeras, and protein-nucleic acid complexes like the CRISPR-Cas9 system [15,16,17,18,19,20,21,22].

Molecular dynamics simulations, starting from experimental structures of protein-nucleic acid complexes, remain the most common practice to give insight into local conformational changes and protein-DNA interaction networks. While many such simulations remain stable around the initial conformation, some systems are known to undergo a rapid conformational change into a noncanonical due to remaining force field limitations [23]. However, we lack starting structures for many systems, as there are less than 10 K protein-nucleic acid structures in the protein data bank [24]. Even when an initial structure is known, sampling large conformational changes is not always possible, despite the current supercomputer resources available. In such cases, combination with other modeling tools might be the only way to discover new states and get insight into the mechanisms of action [25]. The number of modeling tools is greatly reduced with respect to the protein simulation field. On one hand, this is due to the limited portability of enhanced sampling strategies for nucleic acid systems [23,26]. On the other, docking strategies for predicting protein-nucleic acid complexes are not as mature for protein-nucleic acid systems as they are for the protein field [27,28,29,30,31]. This is partly due to the limitations in scoring functions to handle highly charged systems as well as due to the difficulties in modeling nucleic acid conformational changes during binding. Studies like those conducted by Haddock [30,32,33] have incorporated DNA flexibility through normal modes but have not yet taken advantage of the sequence-dependent deformation profiles of DNA.

Simulations of nucleic acid systems expand scales of different lengths, with mesoscopic and coarse-grained models bridging between atomistic simulations and relevant length-scales in the cell [34,35,36,37]. In recent years, several approaches involving coarse-grained and multiscale simulation strategies have been developed to combine proteins and nucleic acids [38,39,40,41]. Our own efforts contributed to the development of a faster generalized Born implicit solvent that can be used for atomistic simulations of proteins and nucleic acids [42], and more recently, these models were combined with the MELD (Modeling Employing Limited Data) approach [43,44] for the simulation of protein-DNA complexes [45].

In this work, we focus on Bacterial DNA polymerase, a complex machinery comprising several proteins and domains. The β subunit is a homo-dimer ring clamp that slides onto the DNA ahead of the replication fork and has multiple interaction points for other proteins. Its nonsequence-specific interactions with DNA and the wide 30 Å to 35 Å opening allow easy sliding. The β subunit plays two critical roles: it eliminates any need for DNA interaction and recognition by the polymerizing subunits, and it prevents early dissociation of the polymerase, i.e., improves processivity [46,47,48]. While the clamp is capable of spontaneous opening and closing, the frequency and lifetime of this phenomenon are low. To accelerate this rare event, a clamp loader will open the ring and load it onto the DNA by consuming energy. Once loaded, other pieces of the polymerase apparatus are recruited [49,50]. In humans, the equivalent of the bacterial β clamp is called proliferating cell nuclear antigen (PCNA). It is a homotrimeric, six-domain ring with <10% sequence similarity to the prokaryotic subunit [51]. Studies of the PCNA homotrimer by molecular dynamics approaches suggest that the clamp loader only stabilizes the open state but does not change the rate of opening events [52]. Experimental studies on the bacterial β-clamp indicate that this is a more stable structure that requires the clamp loader to promote opening events [49,50]. The fundamental differences between the eukaryotic and prokaryotic clamps make it a suitable target for antibacterial research.

**Figure 1:**
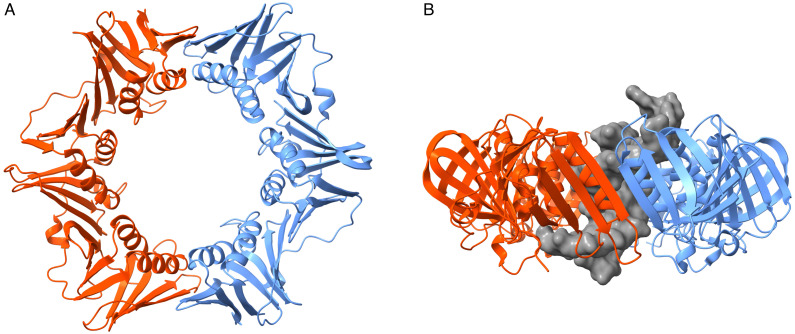
The homo-dimer clamp in its free and DNA-bound states. (**A**). Top-down view of the unbound clamp with the two subunits shown in blue and orange (PDB ID: 1MMI). (**B**). Side view of the clamp bound to an oligonucleotide shown as a gray surface representation (PDB ID: 3BEP).

In this study, we were interested in identifying strategies to sample and identify open states of the β-clamp that may be relevant to the loading of DNA into the clamp. We simulated the bacterial clamp in its free and DNA-bound forms in all-atom (AA) and coarse-grained (CG) representations of the system. We ran implicit and explicit solvent simulations at room and melting temperatures for both representations of the system using a conventional Molecular Dynamics (cMD) approach. Additionally, we employed the MELD enhanced sampling approach using an atomistic representation of the system in implicit solvent. The initial states corresponded to X-ray crystallography structures (PDB codes 1 mmi (unbound) and 3 bep (with DNA), see Figure 1). Our simulation results highlight the difficulty associated with modeling large conformational transitions for protein-DNA systems as well as demonstrating the ability to identify states beyond what cMD can simulate. We conclude that these states can then be used to more accurately describe the systems (all atom simulations in explicit solvent).

## 2. Materials and Methods

### 2.1. All Atom Simulations

The ff19SB [53] force field was used for the description of proteins and the parmBSC1 force field [10] for DNA. For explicit solvent simulations, the structures were solvated in a periodic octahedral box using the OPC water model with 20 Å maintained between the edge of the box and the solvated macromolecules [54]. K${}^{+}$ and Cl${}^{-}$ ions [55] were added to neutralize the system’s charge, and 150 mM concentration similar to physiological conditions was employed [56]. For implicit solvent simulations, the structures were placed in a nonperiodic space with no solute–solute interactions. The GBneck2 solvent model [42,57] was paired with the mbondi3 radii set. The nonbonded interaction cut-off was set to 999 Å for the implicit solvent, and the salt concentration was similarly set to 150 mM. All systems were minimized using four descending restraint weights: 25, 20, 15, and 5 kcal/mol. The first 2000 steps of each minimization stage applied the steepest descent algorithm [58] with the last 2000 steps of each minimization stage applying the conjugate gradient algorithm [59]. Explicit systems were then gradually heated to two temperatures, 298.15 and 368.15 K, using the Langevin thermostat for 50 ps. Then, the system equilibrated under NVT conditions for 950 ps with a time step of 1 fs and a collision frequency of 2 ps${}^{-1}$. They were then equilibrated for 2 ns under NPT conditions to stabilize pressure at 1 bar using the Berendsen barostat with 1 ps of relaxation time [60]. SHAKE was used to constrain bonds involving hydrogens [61], and a 10 Å cutoff was used to approximate long-range electrostatic interactions using the particle mesh Ewald method [62]. Implicit solvent systems began production runs after heating. For the first nanosecond of equilibration, 5 kcal/mol restraints were applied. For all systems, production was run using the GPU-enabled version of *pmemd* for 1 μs [5]. Simulation protocols were run in triplicate for each system.

### 2.2. Coarse-Grained Simulations

AA structures were mapped to coarse-grained models using the cgconv tool developed with the SIRAH 2.0 force field [39,40]. For the explicit solvent set up, input structures were solvated in an octahedron box using the WAT FOUR (WT4) water model with a physiological concentration of 150 mM Na and Cl ions in a coarse-grained representation and a clearance of 20 Å from the boundaries of the box [63]. Heating to 298 or 368 K, was done over 100 ps, and for explicit solvent systems, this was followed by 1 ns of NVT and NPT equilibration using the Langevin thermostat (collision frequency of 2 ps) and the Berendsen barostat (coupling constant of 1 ps) [60,64]. Long-range calculations beyond 12 Å were approximated using the Particle Mesh Ewald (PME) [62]. The Hawkins, Cramer, Truhlar (HCT) pairwise generalized Born implicit solvent model (igb = 1) was used for implicit simulation runs to mimic a salt concentration of 150 mM, and no long-range cut-offs were used [65,66]. Production runs spanned 30 μs for explicit solvent systems and 50 μs for implicit solvent systems.

### 2.3. Basics of Replica Exchange Molecular Dynamics and MELD

Replica Exchange Molecular Dynamics is an advanced sampling technique in which several copies (replicas) of an identical system are run in parallel, and the conditions along the different replicas might change (e.g., Temperature or Hamiltonian). At periodic time intervals, swaps between replicas are attempted and accepted according to the metropolis algorithm [67,68]. Through this random walk along the replica conditions, the sampling efficiency is increased. MELD uses an H,T-REMD protocol in which the Hamiltonian is changed depending on (1) how strongly restraints are enforced on top of the potential energy (coming from the force field) and (2) which subset of the restraints is active (see [43,69]).

### 2.4. MELD Setup

For meld systems, initial structures were minimized according to explicit solvent all-atom protocols and then fed into the meld as input files. We used the ff14SBside [70,71] and parmbsc1 [10] force fields to describe the system with the GBneck2Nu implicit solvent [42]. From each initial structure, 50 replicas with a temperature range from 300 to 500 K were constructed. Exchanges were attempted every 50 ps and were accepted or rejected according to the metropolis criteria. In all meld simulations, the tertiary structure of each clamp subunit was loosely restrained to avoid unfolding at higher temperatures by applying a flat-bottom harmonic restraint between any two CA atoms within 8 Å of each other in the initial structure. Two sets of MELD simulations were performed. In the first set, no restraints were added between the two monomers constituting the clamp dimer, which lead to some irreversible dissociation in high-temperature replicas. A second set of simulations enforced distance restraints at one of the two monomer interfaces (see Figure 1). Clamp simulations are summarized in Table 1.

### 2.5. Indicators for Conformational Fluctuations

We used several measurements to monitor the conformational fluctuations in the clamp structure. Backbone RMSD was used as a general indicator of structural similarity to the experimental structure. To monitor any opening event in the interfaces of the two subunits, we measured the distance between two groups of residues located within a pairwise distance of 8 Å (CA-CA). We averaged over the inverse of all such pairwise distances to reduce the effect of a few long distances. Additionally, to keep track of the orientation of the two subunits relative to each other, we monitored the angle between the planes of the two subunits. We picked three CA atoms in each subunit (residues 75, 169, 267 and 441, 535, 633), one near each interface and one around the center of the half-ring. Then, the equation of the plane passing through the three atoms was calculated, and the angle between the two planes was measured. In the experimental structure, the angle was about 172°, as expected given the planarity of the clamp (See Figure S1 for a visualization of these measures).

## 3. Results

The clamp dimer is made up of a homodimer with C2 symmetry. Each monomer is made up of three α + β domains, where helices face inwards defining a ring that slides along the DNA. Long, unstructured loops join the different domains. Both monomers rest on the same plane. Our goal was to benchmark the ability of different sampling strategies to explore the opening of a DNA clamp required for binding DNA. Such an opening motion can be best tracked by following the distances of residues at the two monomer interfaces as well as the angle between the normal of the planes defined by the interface (see methods). We used all-atom and coarse-grained simulations in both implicit and explicit solvents as well as the MELD (Modeling Employing Limited Data) ti enhance the sampling strategy.

### 3.1. Validation of the Methods

While all atom explicit solvent simulations are standard in the field, other types of simulation options have not been as widely tested. Our recent work in using all-atom simulations with implicit solvent (GBNeck2Nu) has been independently tested by the community (see for example [72,73,74]. Studies using the coarse-grained model Sirah for protein-nucleic acids and MELD have been more limited, and hence, a few systems were simulated here.

#### 3.1.1. Coarse-Grained Simulations of Three Transcription Factors Bound to DNA Remain Bound throughout the Simulation Timescale

We first established Sirah’s ability to capture protein-DNA interactions by performing 10 μs unbiased coarsed-grained simulations of three small transcription factors bound to DNA chosen from the HADDOCK benchmark test set [31]: Hyperthermophile Chromosomal Protein Sac7d (PDB ID: 1AZP), Nuclear Intron-encoded Homing Endonuclease I-PpoI (PDB ID: 1A74), and 9-cis Retinoic Acid Receptor(PDB ID: 1BY4). All systems were found to remain bound for the entirety of the sampling time and with a similar pose to the experimentally determined conformation. We used a hierarchical algorithm to cluster the latter 5 μs of the production. For sac7d, all frames in the second half of the simulation formed only one cluster with an RMSD of 8.4 Å. I-PpoI had several clusters with the top three RMSDs being 9.7 Å, 10.0 Å and 10.7 Å and the population fractions being 36%, 30%, and 18%, respectively. Finally, the 9-cis receptor ended up with an individual cluster with an RMSD of 7.25 Å. Figure S2 shows the superposition of these clusters with the corresponding crystal structures. The observed changes were mostly due to protein rearrangements in the loops and tails rather than at the protein-DNA interface, which is in line with benchmark studies conducted by the force field authors [40].

#### 3.1.2. MELD Simulations Capture Multiple Binding/Unbinding Events of Proteins to DNA

**Figure 2:**
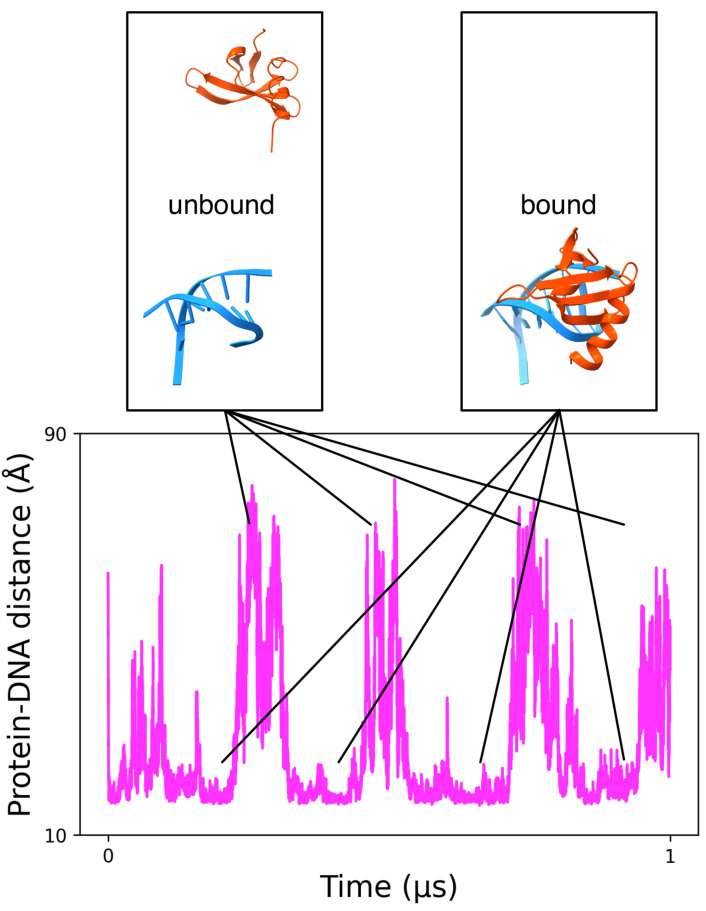
Schematic of the binding/unbinding events explored for a random walker in a MELD simulation of transcription factor binding. The magenta graph shows the distance between centers of mass of the protein and the DNA. Representative insets of the structures sampled are provided above the graph.

MELD has been successfully used to study the binding of small molecules [75], peptides [69,76,77], and proteins [78] to other proteins. Our recent development of an implicit Generalized Born solvent model that is compatible with both proteins and DNA has opened the possibility of simulating protein-DNA binding [45]. These simulations use T-,H-REMD (see methods) to simulate the binding/unbinding process. Hence, information is used to keep the DNA and proteins from denaturing at higher temperatures while allowing them to unbind. We used sparse, noisy, and ambiguous data to guide the proteins towards the DNA with varying force constants. Figure 2 follows a “walker” in the replica exchange ladder as it goes up and down the replica exchange ladder. High RMSD values with respect to the experimental complex represent unbound events, while intermediate and low RMSDs represent misbound and native-bound states. This example shows the ability of MELD to sample large conformational transitions for protein-DNA systems with multiple binding/unbinding events. Furthermore, it shows the ability to recover the correct binding mode in agreement with experiments (see [45] for further details). Using all “walkers” to analyze the binding ensemble (50 different replicas) improved the statistics for binding/unbinding events significantly.

### 3.2. Simulations of the Homodimer DNA Clamp

Simulations were carried out in triplicate for all cases using implicit and explicit solvents, high and low temperatures, and either coarse-grained or all-atom simulations, giving rise to 8 different sets of conditions. Simulations were further done in the presence and absence of DNA (16 types of simulations). Figures S3–S7 summarize the overall behavior of the system under different conditions. Figures S8 and S9 summarize the behavior at the dimer interface. A brief description of each system is given below.

### 3.3. All-Atom Simulations of the Clamp in Explicit Solvent Showed a Stable Clamp Structure

**Figure 3:**
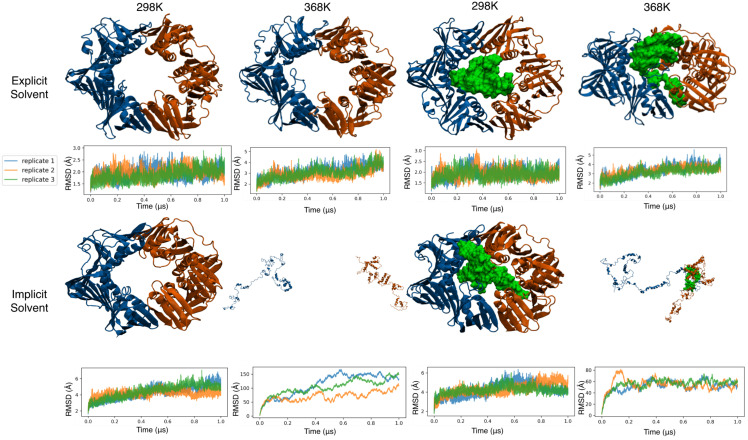
Final structure sampled for all-atom systems along with the backbone RMSD of the clamp for the triplicate samples tested for each combination of temperature and solvent. Snapshots were taken of the first replicate. The first subunit is shown in orange and the second is shown in blue, both as cartoon representations. For simulations in the presence of DNA, a green surface representation is used.

The clamp structure and its two domains remained stable with minimal deviation from the crystal structure in all-atom systems that were run in explicit solvent at room temperature. The two subunits remained bound at both interfaces without sampling any opening event. Furthermore, the two monomers remained in the same plane, and the individual domains in each monomer conserved their structures and relative orientations to each other. Since this force field combination is the most accurate, we used these simulations as benchmarks for all other setups. The RMSD for the bound and unbound clamp and the RMSD of its individual subunits remained mostly below 3 Å (see Figures S5 and S6). At high temperatures, the whole ring and individual subunit RMSDs showed a rising trend, reaching around 6 Å by the end of the microsecond-long sampling period (see Figure 3). The higher RMSD is in agreement with increased thermal fluctuations at this temperature. As in the lower temperature runs, no opening event was observed in the clamp at either dimer interface in any of the triplicate runs—either in the presence or absence of DNA. This behavior agrees with the expected long time-scale for such opening events.

### 3.4. All-Atom Simulations of the Clamp in Implicit Solvent Accelerate Heat Denaturation

As expected, the use of an implicit solvent led to higher RMSD values (see Figure S3). This was in part due to the increased sampling efficiency in the absence of an explicit solvent [79] and in part due to the lower accuracy of the force field under implicit solvent conditions. At 298 K, the fluctuations stabilized at around 6 Å in the native structure within the first 600 ns and remained there for the length of the simulation. The increased RMSD did not change the overall topology of the system and was mostly due to increased fluctuations in long loop regions connecting the neighboring domains of each subunit (residues 109–126 and 235–255 in each subunit, see Figure S4). Simulations conducted under high temperatures presented a different picture: the two monomers rapidly dissociated, and the internal structures of the domains in each monomer were lost. This lower stability in implicit solvent was expected. However, in the presence of DNA, the two monomers nucleated around the DNA as the domains lost their original structures. Overall, these simulations did not lead to reversible opening of the clamp being observed. A canonical closed conformation was observed under lower temperatures, and a dissociated denatured state was observed under higher temperatures (see Figure 3).

### 3.5. Coarse-Grained Simulations of the Clamp Spontaneously Sample Open States

**Figure 4:**
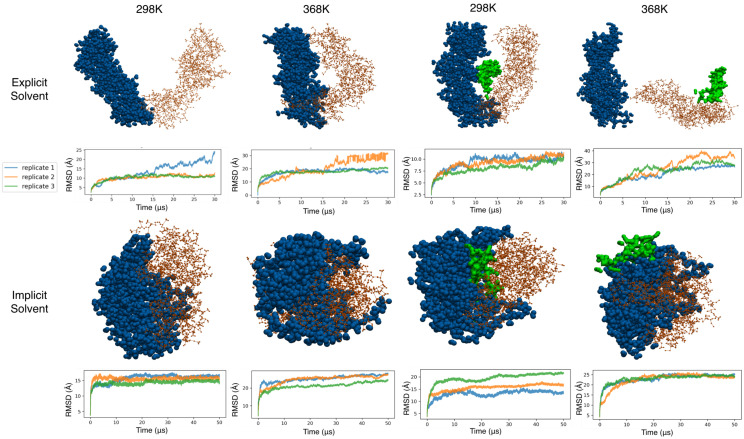
Final structure sampled for coarse-grained systems along with the backbone RMSD of the clamp. The first subunit is shown as orange CPK, and the second is shown as blue VDW spheres. DNA is represented as a green surface where applicable.

In our study, the simulation of the system with the Sirah coarse-grained potential showed very different behavior. Regardless of the presence/absence of DNA, we saw early clamp opening events during the simulations in both explicit and implicit solvents. These opening events were not reversible in the simulated timescale, leading to structures being far from the experimental state (see Figure 4). Interestingly, each of the three domains in each monomer maintained their structures, but the hinge region between domains led to a relative rotation of the individual domains with respect to the experimental structure. The cumulative effect of this rotation between domains led to large overall conformational changes in each monomer (∼12 Å), and the coplanarity of the two monomers was lost. While the monomers had smaller RMSD values in the presence of DNA (∼10 Å), the overall conformational change of the dimer was amplified as the two monomers wrapped around the DNA. These observations were amplified under high temperatures. There was a marked difference between simulations conducted in implicit and explicit solvents, with the former producing more compact structures. This difference in behavior likely arose from an imbalance in the force field in the implicit solvent. In summary, Sirah coarse-grained simulations are better suited for coarse-grained explicit solvent in this system. The individual domains of the proteins and the DNA structure were shown to be well maintained, but the hinge regions seemed to be more flexible than the all-atom counterpart, leading to irreversible opening events in the simulated timescale.

### 3.6. MELD Samples Show Frequent Spontaneous Opening and Closing of the Clamp

**Figure 5:**
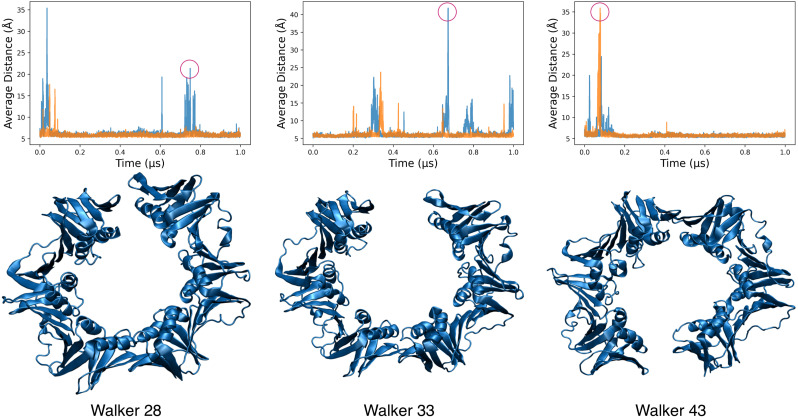
Snapshots from three random walkers of the unbound clamp with no restraints on any of the interfaces. Corresponding plots of the average distances of the interfacing residue groups are shown on top of each structure, in which orange and blue correspond to the two joints.

For this large system, we used 50 replicas to extend the H,T-REMD to high temperatures, which induced opening while maintaining the tertiary structure. We tracked the opening as in other cases through the distance between groups of residues at the interface. In the “unrestrained” simulations where the two joints were free to open, we observed that half of the walkers showed frequent opening and closing events for the unbound clamp (See Figure 5). The opening and closing usually happened at one joint at a time and ranged from around 15 Å to almost 80 Å (see Figure S10). Contrary to the coarse-grained simulations, the opening events separated the two domains close to the plane of the clamp with deviations as high as 30° off the plane (see Figure S11). We observed opening events on either of the two monomer interfaces as expected, due to symmetry. However, due to the higher temperature replicas, many walkers eventually sampled dissociated states that were not relevant for the current work, as they were irreversible (the two monomers diffused away from each other). Such behavior led to lower exchanges in the H,T-REMD between dissociated and assembled states (see Figures S12 and S13). In the DNA-bound systems, dissociated dimers tended to nucleate around the DNA, most likely due to an overstabilization of protein-DNA interactions (see Figure S14). To reduce such dissociation events, we performed new MELD simulations to restrain one of the two interface regions. In this situation, exchanges in the H,T-REMD were more efficient (see Figures S12 and S13), and multiple opening/closing events were observed at the unrestrained interface (see Figures S15 and S17). These opening events took place on the nanosecond timescale, with higher frequency as the temperature increased, but their lifetime was fairly short (picosecond timescale). Looking at the ensembles at different temperatures (see Figure S16), we observed no opening events at lower temperatures, and opening events capable of incorporating double-stranded DNA only occurred in the higher replicas.

**Figure 6:**
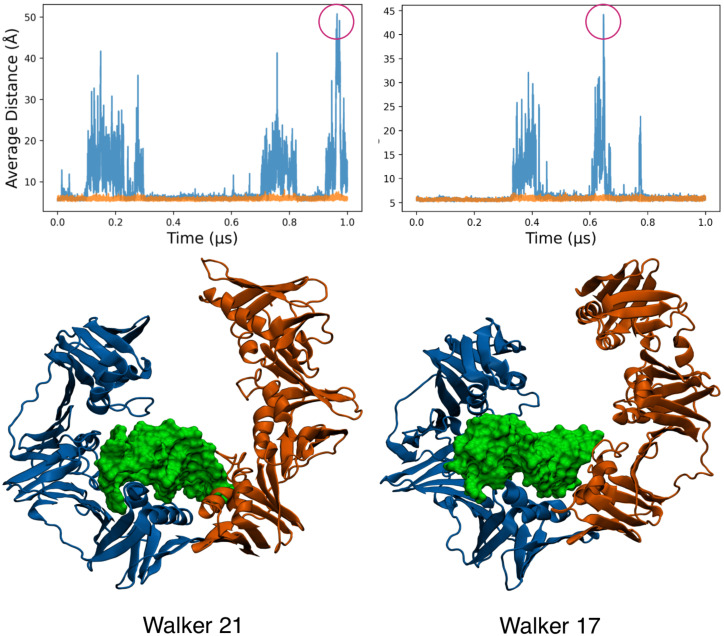
Snapshots from two random walkers in the MELD simulations for the DNA-bound clamp and restraints on one interface. The protein monomers are shown in blue and orange, and DNA is shown as a green surface. The insets show the frequency of opening events for each of the two walkers.

In these restrained simulations, for 36 of the walkers, we observed reversible ring opening in the unbound clamp at the unrestrained interface. In the presence of DNA, we found a smaller number of walkers presenting reversible opening events (6/50) of the protein dimer (see Figure S18). However, this seems to have been due to the overstabilitization of an alternative structure in which the monomers increased the interface area between protein and DNA (35/50 replicas, see Figure S14B). In the few walkers that remained conformationally similar to the unbound clamp, open states took longer to close. Additionally, in such walkers, the DNA maximized its interface with one subunit by establishing interactions with the central domain of the subunit. This led to reduced interactions with the other subunit, allowing it to dissociate at the unrestrained joint. We observed major interactions in which an α helix from the domain adjacent to the restrained interface went deep into the major groove, while coils from the middle domain were inserted into the minor groove (see Figure 6).

We conclude that MELD simulations are an efficient sampling strategy for this type of modeling where the goal is to sample conformations that are experimentally detected but for which there are no structures. However, the clear limitations in the choice of implicit solvent for these systems with many possible interacting regions between protein and DNA can lead to overly compact structures. Thus, for quantitative understanding of the observed opening/closing events and lifetimes, the user would need to solvate the structures sampled here in explicit solvent and carry out further studies (e.g., using end-point techniques such as umbrella sampling [80]).

### 3.7. Comparison of Sampling Efficiency

The β clamp is a relatively large system with 732 protein amino acids. Simulating the system in implicit solvent conditions led to ∼12,000 atoms, which became inefficient due to the $N^{3}$ scaling in implicit solvent. Explicit solvation significantly increased the number of particles in the system, but thanks to the Particle Mesh Ewald, this scale of the simulation was Nlog(N), where *N* is the number of particles (∼179,000 atoms). On an RTX2080Ti GPU, 100 ns of the bound systems took approximately 60 h to calculate for all-atom explicit systems and 73 h for implicit ones. In the absence of DNA, simulations were faster (56 h for explicit and 65 h for implicit solvent). Thus, for cMD simulations of large systems, the more accurate explicit solvent is a better choice. Unfortunately, generalized ensemble methods based on REMD, like MELD, require a larger number of replicas with an increased number of particles, limiting the use of explicit solvent in MELD simulations. Coarse-grained simulations contained ∼18,500 atoms (explicit solvent) or 3600 atoms (implicit solvent), and simulations took 80 and 30 min, respectively, for 100 ns of sampling. MELD jobs of the bound system took around 10 h for every 100 ns. All simulations were done on our local supercomputer, with production running on GTX 2080Ti GPUs. For MELD jobs, we used 50 replicas, with each replica requiring a single GPU (every MELD job required 50 GPUs.) Each AMBER simulation was performed on a single GTX2080Ti.

## 4. Discussion

The two Achilles’ heels of molecular simulations are force fields and sampling. The relevance of length scales is their importance in DNA genome packing, nucleosome formation, or more detailed binding mechanisms, which require different modeling approaches, from genomic and mesoscopic level simulations to coarse-grained and all-atom simulations. While all atom simulations in explicit solvent are more physically accurate, they cannot scale to sample meaningful timescales in large molecular assemblies. Different levels of approximation can provide structural insight to test hypotheses that drive research, together with experimental evidence. Our interest in this work lies at the boundary where coarse-grained and atomistic models meet: atomistic models can provide the right details and are limited by sampling, and more coarse-grained models can sample important states that are not accessible to atomic simulations but might also stabilize noncanonical states. Many advanced simulation methods, and even adaptive Markov State Models [81,82], benefit from identifying end-states that focus on all-atom sampling in relevant regions of the energy landscape. In such scenarios, higher efficiency sampling strategies (Coarse graining, implicit solvent, MELD or others) can identify states relevant to the system for posterior use with a more accurate force field. In this work, we used a combination of different approaches to identify open clamp states that are rare and transient when they occur spontaneously. In particular, the clamp in the absence of DNA is experimentally known to undergo reversible opening events with a higher frequency than when DNA is present. In bacteria, the clamp-DNA complex requires enzymatic unloading [83]. These opening events take place over long simulation timescales that we did not observe in all-atom explicit solvent simulations. Using more coarse methods, we observed three types of opening events—two of them were irreversible and not meaningful for our purpose, while the third case led to multiple opening/closing events that should be studied further.

As expected, the simulations using atomic models with explicit solvent were not able to sample these slow events on the microsecond time scale, and they remained stable even at high temperatures. Implicit solvent simulations at an all-atom level of resolution showed lower stability as the temperature increased. This was expected, and dissociation of the two monomer units was consistent. In the presence of DNA, the simulations maximized the interactions between protein and DNA, even at low temperatures. Despite recent advances in implicit solvent models for nucleic acids, the quality remained lower than when simulating protein systems—the difficulties in modeling highly charged systems with implicit solvation models are well known and are difficult to address. Over the lifetime of biomolecular simulations, DNA simulations have lagged behind their protein counterparts by about 10 years due to their highly charged interactions, which made unrestrained simulations unviable prior to the introduction of the Particle Mesh Ewald approach [84]. Even with these corrections, force field inaccuracies limited the breaching of timescales accessible to protein systems [7,85] until recently [8]. Challenges with improving implicit solvent descriptions and developing benchmark tests to identify possible imbalances between protein and nucleic acid force fields remain. These force field were derived in isolation from each other, with a few recent studies indicating that close contacts between DNA phosphate groups and positively charged protein residues [86,87,88,89,90] (arginine and lysine) lead to overly strong electrostatic interactions, and some groups have suggested the existence of deviations from the standard Van der Waals combination rules when simulating protein-nucleic acid systems [89,91]). While there is still debate about this behavior in explicit solvent, our simulations in implicit solvent using either the Sirah coarse-grained model or all-atom simulations showed a marked preference for compact structures and overly stable protein-DNA interactions. These issues increased as the available protein-DNA interaction surface increased (larger protein and DNA systems). The incorporation of data and restraints can help in these cases to compensate for force field deficiencies. Using this strategy within the MELD approach has already been successful for modeling protein systems [92]. Our use of restraints within MELD maintained the structures of the individual protein domains in the REMD as well as the double-stranded nature of the DNA, but did not change the protein-DNA interactions or monomer-monomer interactions in the clamp. Effectively, this led to multiple replicas dissociating at high-replica indices, with few exchanges occurring between the lower and upper replicas. Despite this, the lower replicas remained stable, with multiple opening events being observed at either protein-protein interface. Restraining one of the two interfaces improved the exchanges between all replicas, allowing better statistics to be collected for the opening events. MELD atomistic simulations support a reversible (and infrequent) opening mechanism with a very short lifetime with small deviations from the plane of the clamp dimer. Coarse-grained simulations, on the other hand, supported irreversible, out-of-plane opening events. Future work will explore the opening/closing mechanisms and lifetimes using explicit solvent and adaptive sampling strategies [93] seeding from the structures sampled in this work.

Despite the above limitations, these tools can provide useful insights that can be used when modeling large conformational changes in protein-nucleic systems. For example, docking approaches are typically used to predict small molecule and protein binding but are not suitable for sampling the large conformational changes of protein-DNA binding, as they ignore sequence-dependent properties of DNA [27,28,29,30,31], and their scoring functions are less reliable for highly charged systems. Both Sirah and MELD methods, when using implicit solvent, exhibit a sequence-specific response [39,45] and can tackle deformations beyond the harmonic regime. The combination of states discovered from these techniques with enhanced sampling approaches using end-point techniques or Markov State Models in explicit solvent are attractive solutions that may complement our structural and dynamical understanding of protein-DNA complexes. Further efforts that combine MELD with coarse-grained force fields and take advantage of hybrid multiscale approaches will further increase capacity to model nucleic acid complexes. Promising force field developments coming from machine learning will also provide solutions to issues arising from phosphate-protein interactions.

## 5. Conclusions

We set out to study the suitability of different sampling approaches including coarse-grained and enhanced sampling (MELD) to capture transient and infrequent opening events in an *E. coli* β-clamp. Our simulations successfully captured two distinct opening mechanisms (out-of-plane and in-plane). The former was irreversible in the timescale studied, in conflict with previous data, while the in-plane opening events captured by MELD agreed with the expected low-frequency events. Our study also identified marked differences in the presence of DNA, likely arising from an imbalance in the implicit solvent. Nonetheless, these sampling strategies provide structural data that can be used as the basis of future studies using adaptive sampling in explicit solvent seeding from the structures identified in this work.

## Figures and Tables

**Table 1:** Details of the bacterial β clamp simulations in this study.

Scale	Solvation	Solvent	State	Temperature (K)	Time (μs)	Replicates
All-Atom	explicit	OPC	bound	298	1	3
				368	1	3
			unbound	298	1	3
				368	1	3
			bound	298	1	3
				368	1	3
			unbound	298	1	3
				368	1	3
	implicit	GBneck2	bound	300–500	1	1
					1	50 replicas
			unbound	300–500	1	1
					1	50 replicas
Coarse Grained	explicit	WT4	bound	298	30	3
				368	30	3
			unbound	298	30	3
				368	30	3
	implicit	HCT	bound	298	50	3
				368	50	3
			unbound	298	50	3
				368	50	3

## Data Availability

Simulation data are available from the authors upon request.

## Supplementary Materials

The following are available online at https://www.mdpi.com/article/10.3390/life12020261/s1, Figure S1: The two subunits of the clamp are shown in orange and blue cartoon. Groups of C atoms used to measure the distance at each interface are shown in purple and green spheres. Imaginary planes that pass through residues 75, 169, 267 and residues 441, 535, 633 are shown in gray and were used to monitor the angle between the two subunits, Figure S2: Three small transcription factor systems sampled for 10 μs with SIRAH in explicit solvent and clustered on the latter half of the trajectory. Clusters are superposed on the reference structure with alignment on the C5X beads of DNA. Green shows DNA in reference conformation, blue is the reference protein. Red is the first cluster from the trajectory, Figure S3: RMSD of the whole clamp protein. The three replicates are shown in blue, orange and green. aa = all-atom, cg = coarse grain, i = implicit solvation, x = explicit solvation, c = cold (298 K), h = hot (368 K), 1,2,3 = replicate number, Figure S4: RMSD of the loop region in non-MELD systems. Notice the increased values at room temperature in all-atom implicit systems compared with explicit systems, Figure S5: RMSD of the first subunit of the clamp. The three replicates are shown in blue, orange and green. aa = all-atom, cg = coarse grain, i = implicit solvation, x = explicit solvation, c = cold (298 K), h = hot (368 K), 1,2,3 = replicate number, Figure S6: RMSD of the second subunit of the clamp. The three replicates are shown in blue, orange and green. aa = all-atom, cg = coarse grain, i = implicit solvation, x = explicit solvation, c = cold (298K), h = hot (368 K), 1,2,3 = replicate number, Figure S7: RMSD of the DNA in the bound systems. “fit” panels show internal RMSD of the DNA while “nofit” panels show it’s reletive RMSD with regards to the clamp. The three replicates are shown in blue, orange and green. aa = all-atom, cg = coarse grain, i = implicit solvation, x = explicit solvation, c = cold (298 K), h = hot (368 K), 1,2,3 = replicate number, Figure S8: Angle between the planes of the two subunits. The three replicates are shown in blue, orange and green. aa = all-atom, cg = coarse grain, i = implicit solvation, x = explicit solvation, c = cold (298 K), h = hot (368 K), 1,2,3 = replicate number, Figure S9: Distance between two groups of residues at each interface (interface 1 and interface 2). The three replicates are shown in blue, orange and green. aa = all-atom, cg = coarse grain, i = implicit solvation, x = explicit solvation, c = cold (298 K), h = hot (368 K), 1,2,3 = replicate number, Figure S10: Distance between two groups of res-idues at each interface (interface 1 and interface 2) for the unbound clamp with no restraints on any of the interfaces. Each plot represents a different walker as they sample through different conditions of temperature and Hamiltonian, Figure S11: Angle between the planes of the two subunits for the unbound clamp with no restraints on any of the interfaces. Each plot represents a different walker as they sample through different conditions of temperature and Hamiltonian, Figure S12: Replica trace for meld simulations. Each replica is represented by a color.

## Abbreviations

The following abbreviations are used in this manuscript:

MELD	Modeling Employing Limited Data
RMSD	Root mean square deviation
AA	All-Atom
CG	Coarse Grain
