# Supplementary material for: Searching for Low Probability Opening Events in a DNA Sliding Clamp
Source: Life (Basel). 2022 Feb 9;12(2):261. doi: 10.3390/life12020261 (PMC8876151; doi:10.3390/life12020261)
Supplement: Supplementary file 1 [file life-12-00261-s001.zip › life-1555733-supplementary.pdf]

life

MDPI

Supplementary Materials

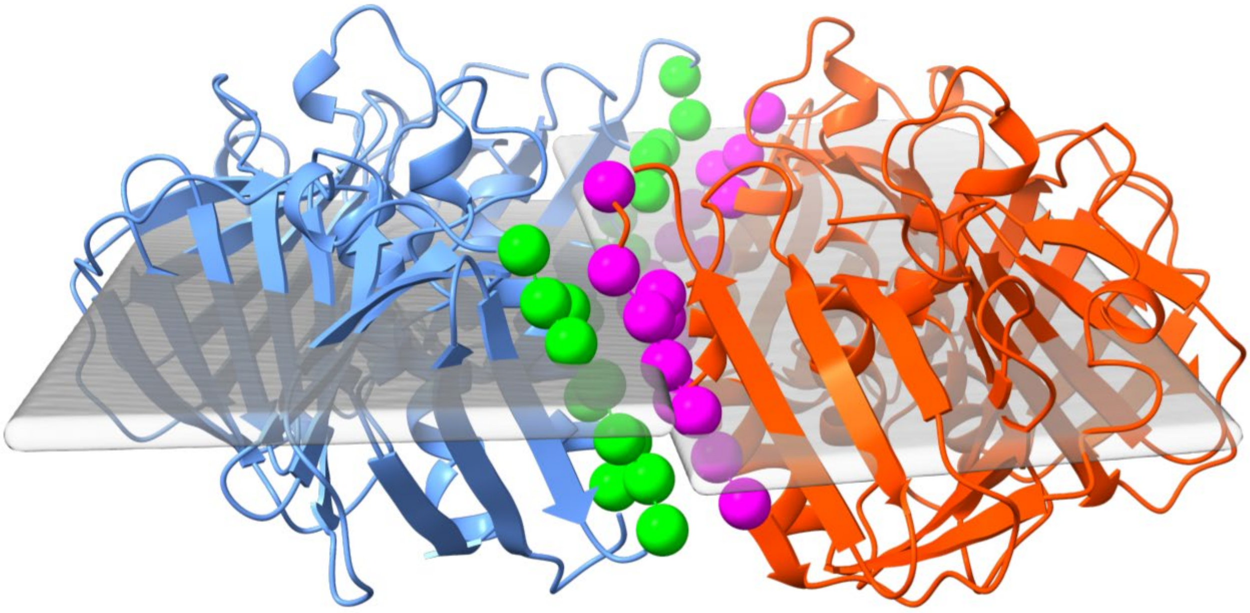

Figure S1. The two subunits of the clamp are shown in orange and blue cartoon. Groups of C atoms used to measure the distance at each interface are shown in purple and green spheres. Imaginary planes that pass through residues 75, 169, 267 and residues 441, 535, 633 are shown in gray and were used to monitor the angle between the two subunits.

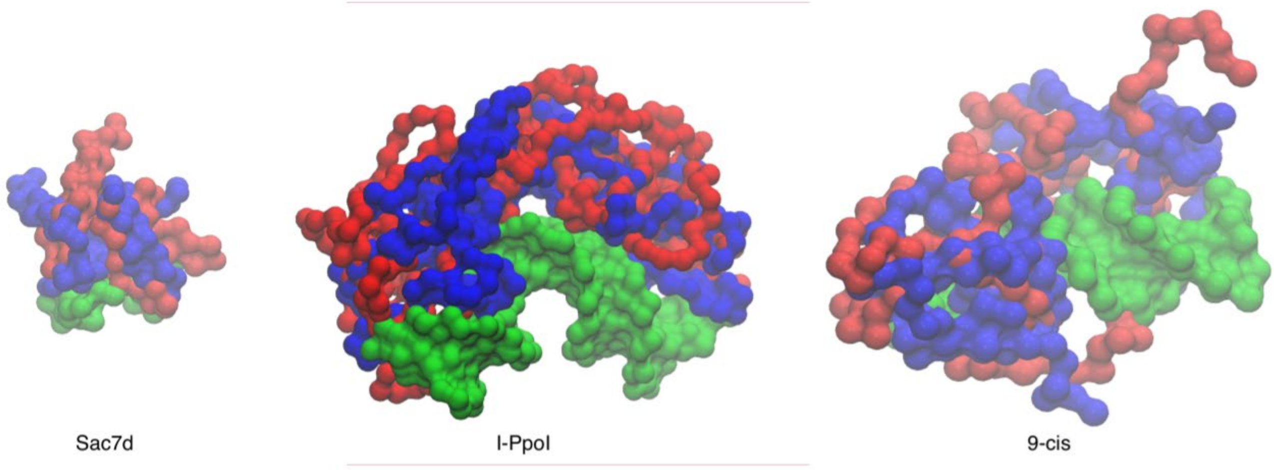

Figure S2. Three small transcription factor systems sampled for 10μs with SIRAH in explicit solvent and clustered on the latter half of the trajectory. Clusters are superposed on the reference structure with alignment on the C5X beads of DNA. Green shows DNA in reference conformation, blue is the reference protein. Red is the first cluster from the trajectory.

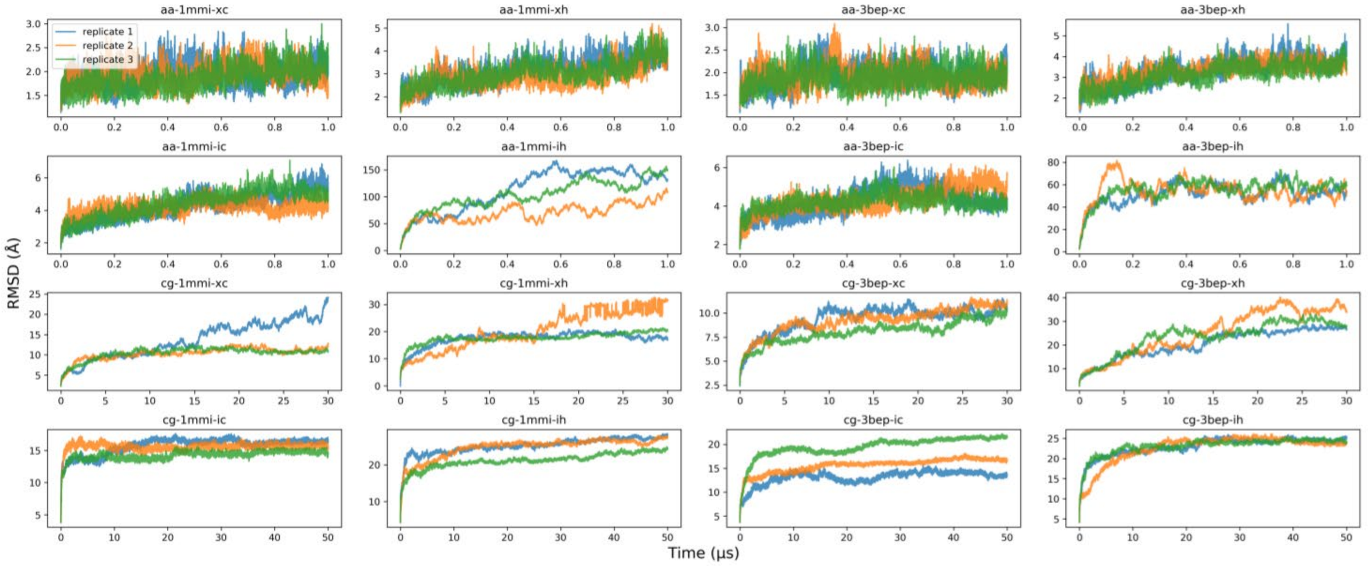

Figure S3. RMSD of the whole clamp protein. The three replicates are shown in blue, orange and green. aa = all-atom, cg = coarse grain, i = implicit solvation, x = explicit solvation, c = cold (298K), h = hot (368K), 1,2,3 = replicate number.

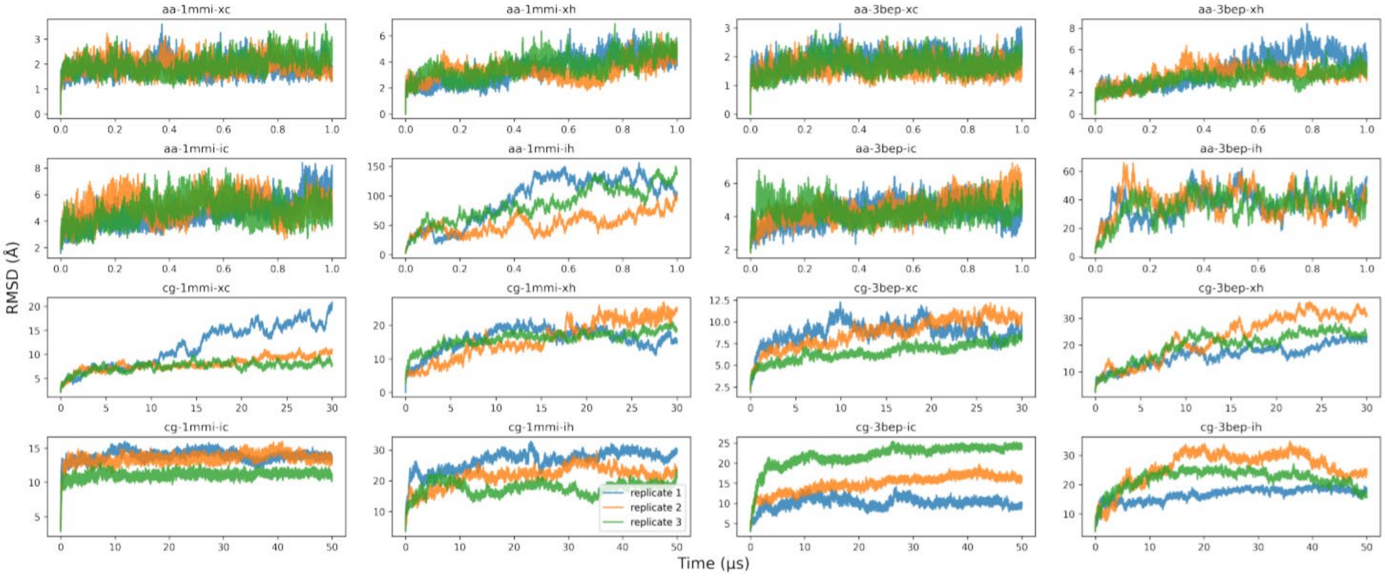

Figure S4. RMSD of the loop region in non-MELD systems. Notice the increased values at room temperature in all-atom implicit systems compared with explicit systems.

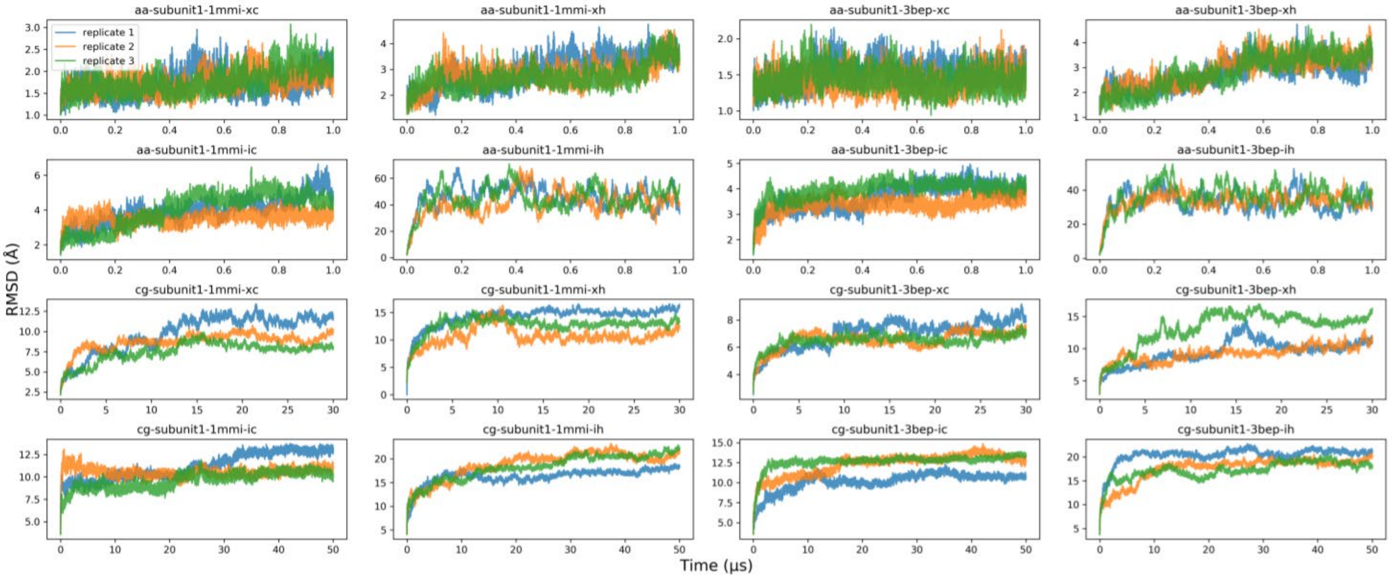

Figure S5. RMSD of the first subunit of the clamp. The three replicates are shown in blue, orange and green. aa = all-atom, cg = coarse grain, i = implicit solvation, x = explicit solvation, c = cold (298K), h = hot (368K), 1,2,3 = replicate number.

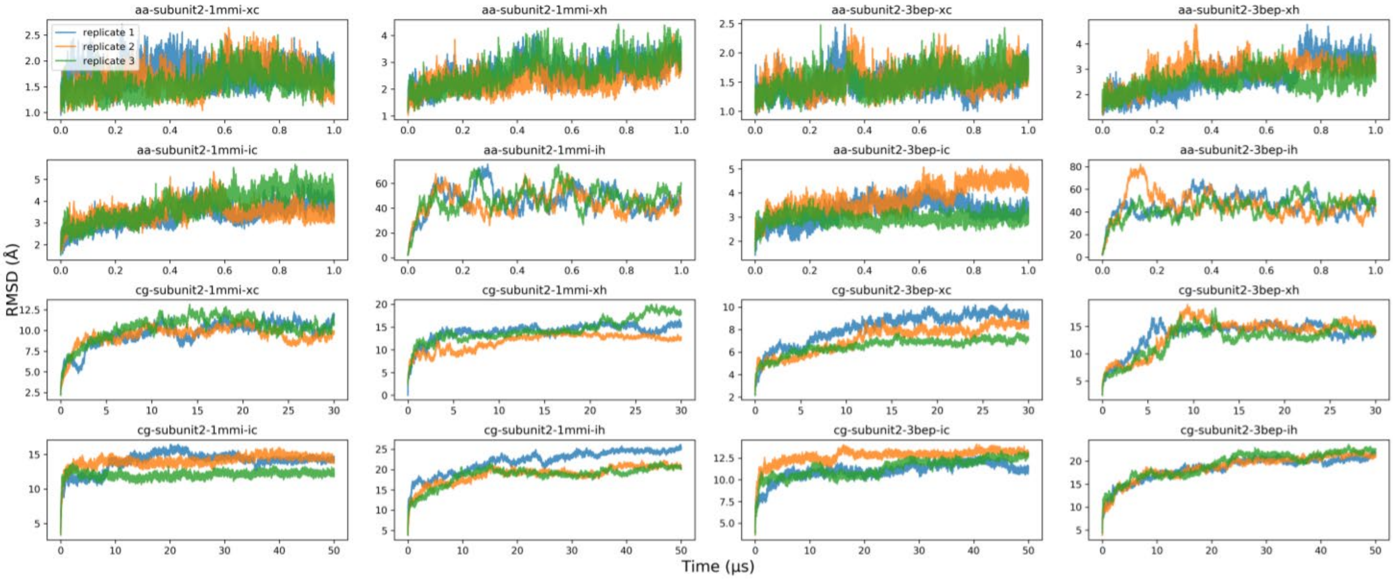

Figure S6. RMSD of the second subunit of the clamp. The three replicates are shown in blue, orange and green. aa = all-atom, cg = coarse grain, i = implicit solvation, x = explicit solvation, c = cold (298K), h = hot (368K), 1,2,3 = replicate number.

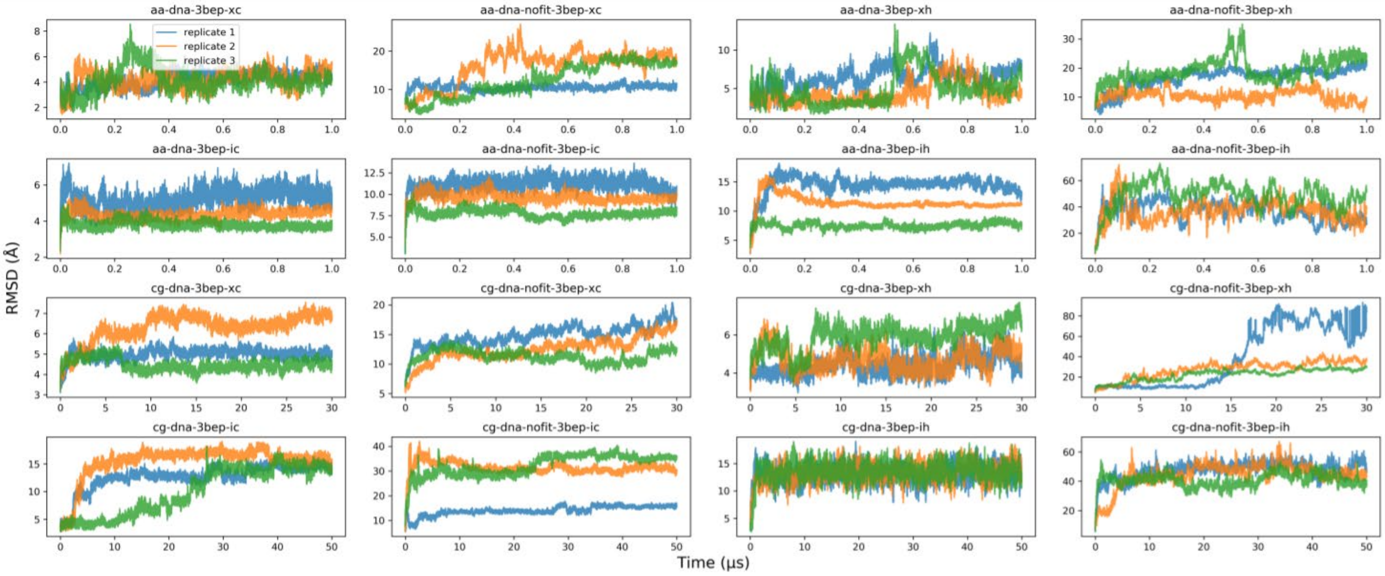

Figure S7. RMSD of the DNA in the bound systems. "fit" panels show internal RMSD of the DNA while "nofit" panels show it's reletive RMSD with regards to the clamp. The three replicates are shown in blue, orange and green. aa = all-atom, cg = coarse grain, i = implicit solvation, x = explicit solvation, c = cold (298K), h = hot (368K), 1,2,3 = replicate number.

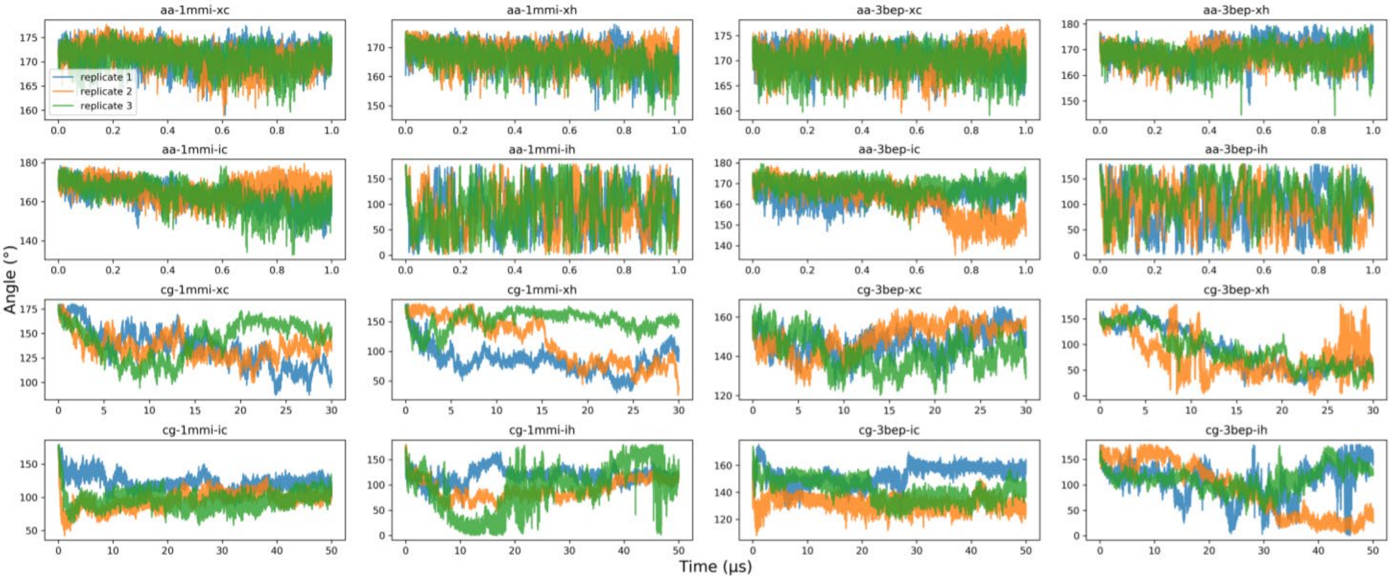

Figure S8. Angle between the planes of the two subunits. The three replicates are shown in blue, orange and green. aa = all-atom, cg = coarse grain, i = implicit solvation, x = explicit solvation, c = cold (298K), h = hot (368K), 1,2,3 = replicate number.

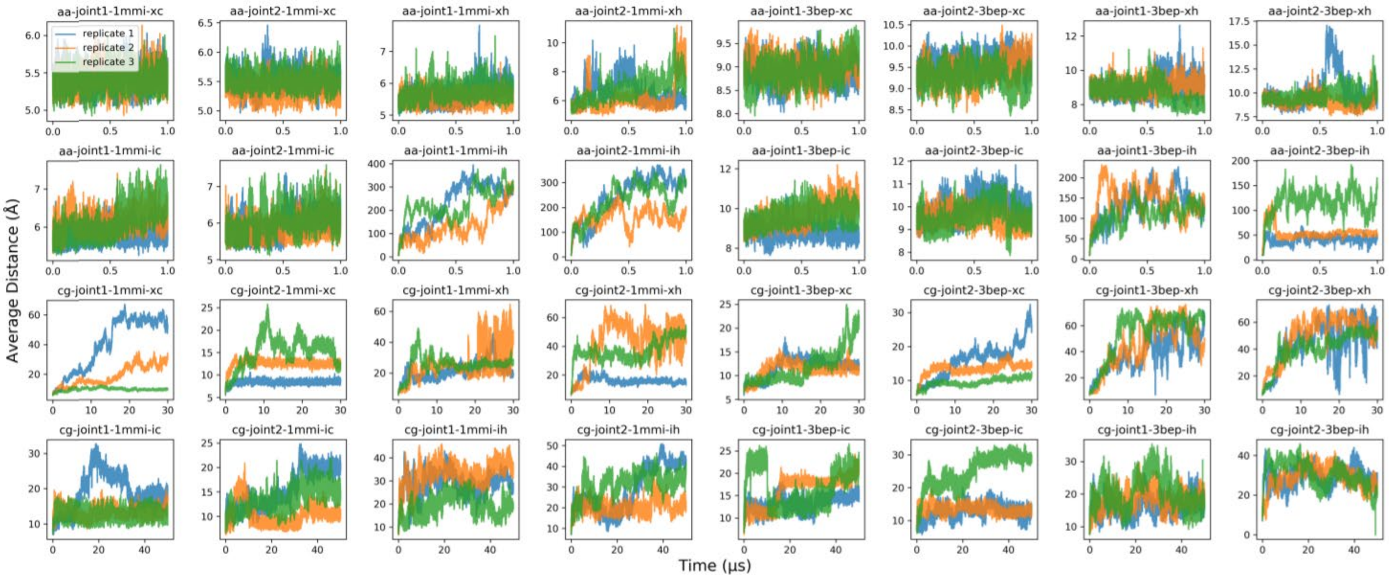

Figure S9. Distance between two groups of residues at each interface (interface 1 and interface 2). The three replicates are shown in blue, orange and green. aa = all-atom, cg = coarse grain, i = implicit solvation, x = explicit solvation, c = cold (298K), h = hot (368K), 1,2,3 = replicate number.

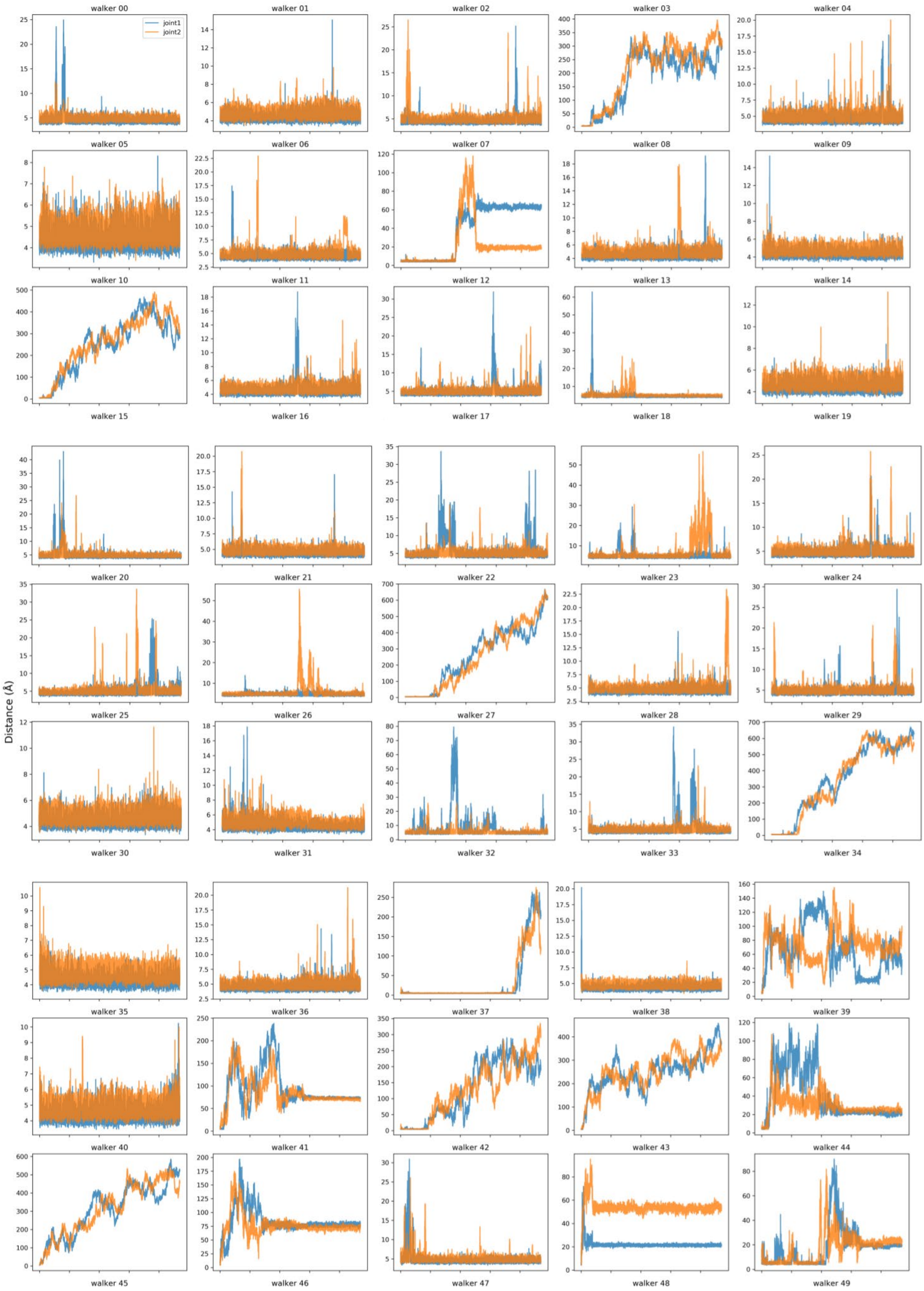
walker 00
joint1
joint2
walker 01
walker 02
walker 03
walker 04
walker 05
walker 06
walker 07
walker 08
walker 09
walker 10
walker 11
walker 12
walker 13
walker 14
walker 15
walker 16
walker 17
walker 18
walker 19
walker 20
walker 21
walker 22
walker 23
walker 24
walker 25
walker 26
walker 27
walker 28
walker 29
Distance (Å)
walker 30
walker 31
walker 32
walker 33
walker 34
walker 35
walker 36
walker 37
walker 38
walker 39
walker 40
walker 41
walker 42
walker 43
walker 44
walker 45
walker 46
walker 47
walker 48
walker 49

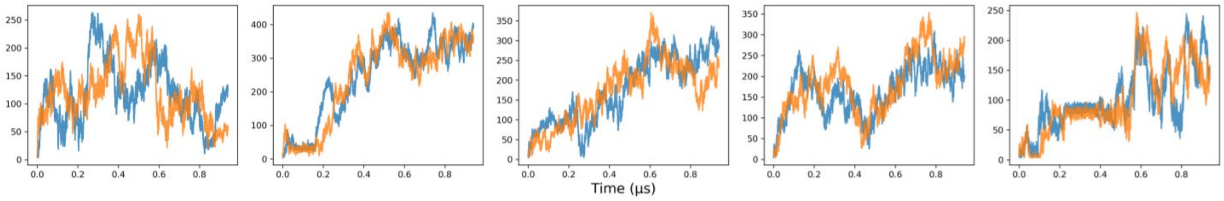

Figure S10. Distance between two groups of residues at each interface (interface 1 and interface 2) for the unbound clamp with no restraints on any of the interfaces. Each plot represents a different walker as they sample through different conditions of temperature and hamiltonian.

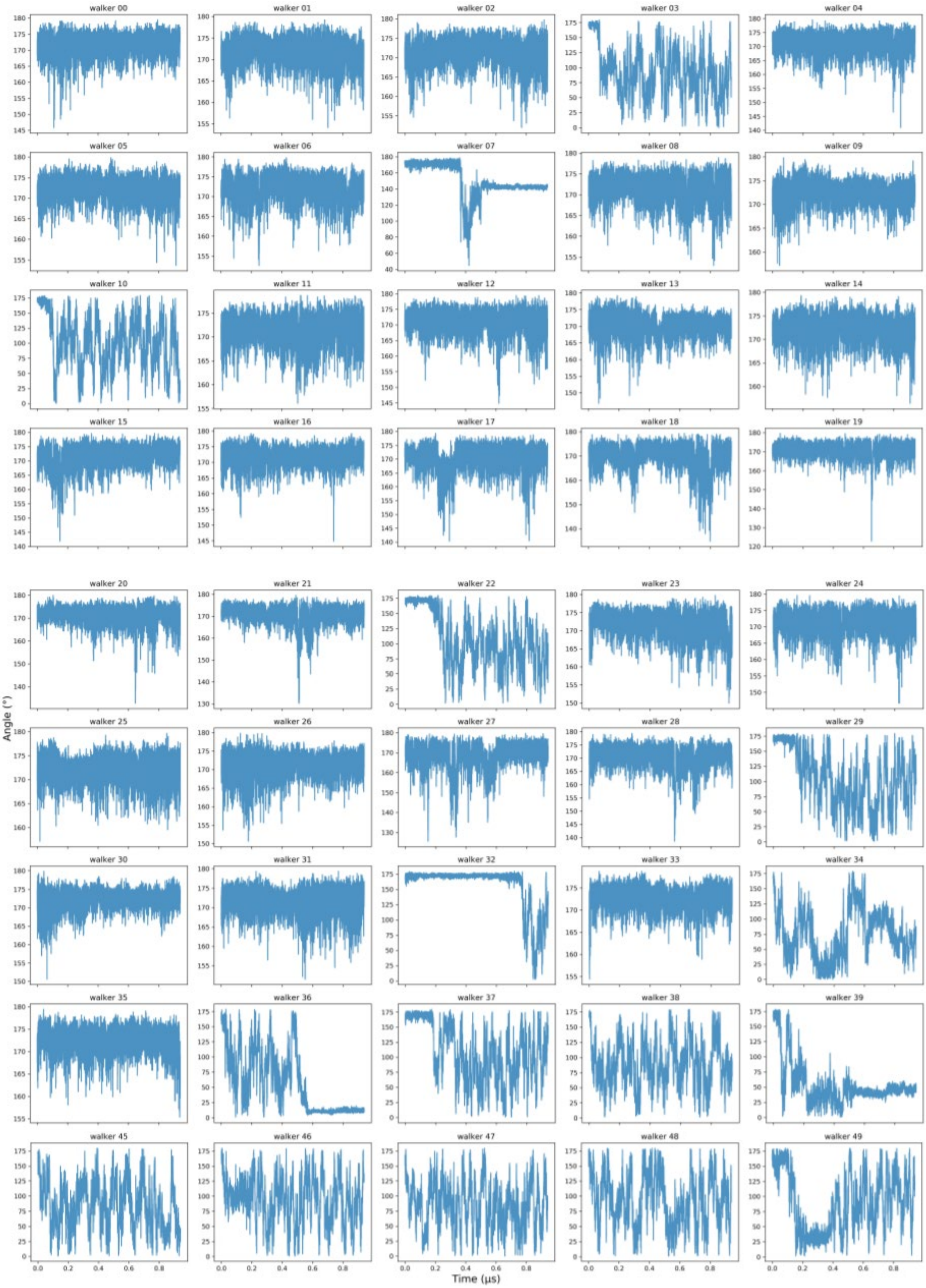

Figure S11. Angle between the planes of the two subunits for the unbound clamp with no restraints on any of the interfaces. Each plot represents a different walker as they sample through different conditions of temperature and hamiltonian.

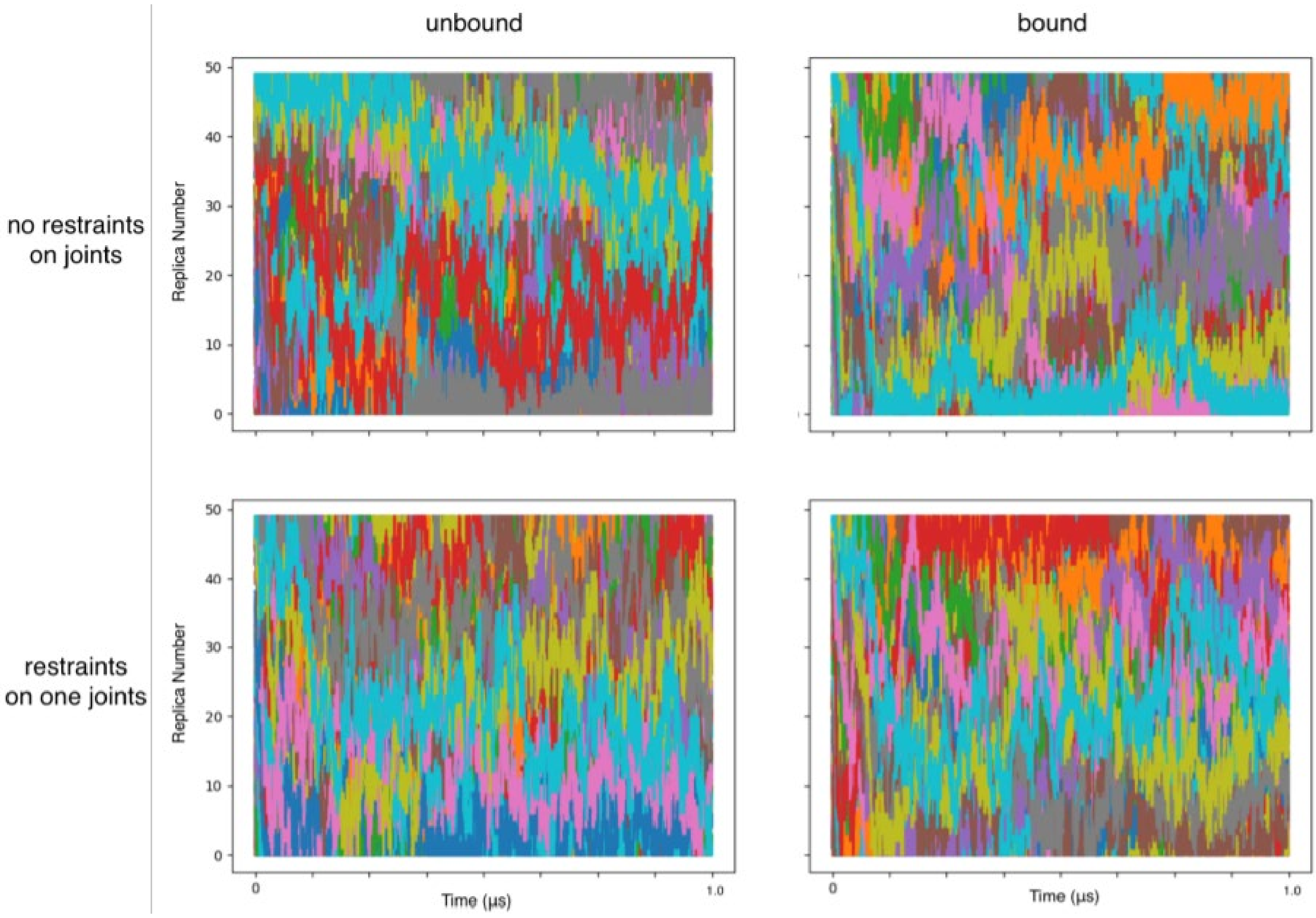

Figure S12. Replica trace for meld simulations. Each replica is represented by a color.
